# Atypical/Nor98 scrapie in the Basque Country: a case report of eight outbreaks

**DOI:** 10.1186/1746-6148-6-17

**Published:** 2010-03-26

**Authors:** Ana B Rodríguez-Martínez, Joseba M Garrido, Sonia Maza, Leyre Benedicto, Mariví Geijo, Nieves Gómez, Esmeralda Minguijón, Sylvie L Benestad, Ramón A Juste

**Affiliations:** 1 Department of Animal Health. Neiker-Tecnalia, 48160 Derio. Bizkaia. Spain; 2 National Veterinary Institute, Department of Pathology, Postboks 750 Sentrum. 0106 Oslo. Norway

## Abstract

**Background:**

Since 2002, an active surveillance program for transmissible spongiform encephalopathy in small ruminants in European Union countries allowed identification of a considerable number of atypical cases with similarities to the previously identified atypical scrapie cases termed Nor98.

**Case presentation:**

Here we report molecular and neuropathological features of eight atypical/Nor98 scrapie cases detected between 2002 and 2009. Significant features of the affected sheep included: their relatively high ages (mean age 7.9 years, range between 4.3 and 12.8), their breed (all Latxa) and their PRNP genotypes (AFRQ/ALRQ, ALRR/ALRQ, AFRQ/AFRQ, AFRQ/AHQ, ALRQ/ALRH, ALRQ/ALRQ). All the sheep were confirmed as atypical scrapie by immunohistochemistry and immunoblotting. Two cases presented more PrP immunolabelling in cerebral cortex than in cerebellum.

**Conclusions:**

This work indicates that atypical scrapie constitutes the most common small ruminant transmissible spongiform encephalopathy form in Latxa sheep in the Spanish Basque Country. Moreover, a new genotype (ALRQ/ALRH) was found associated to atypical scrapie.

## Background

Since 2002, an active surveillance program for Transmissible Spongiform Encephalopathy (TSE) in small ruminants has been implemented in European Union countries. As a result of this program, an atypical type of scrapie different from classical scrapie (CS) and similar if not identical to Nor98 identified in Norway [1] was detected in most of the European countries.

CS is the traditional form of TSE affecting small ruminants, which was first detected in England around 1730 and thereafter in Germany and France [1-3]. Since then, the spread of the disease happened mainly because of the commerce and movement of sheep incubating the disease [4]. Nowadays, the disease is present in many countries of the European Union, as well as in Canada, the United States of America, Brazil, Ethiopia and Japan [5-9]. Only Australia and New Zealand are currently considered "scrapie free" since they have successfully eradicated the classical form of the disease [5]. CS is characterized by transmission under natural conditions causing localised outbreaks with generally a high number of animals affected in certain geographical areas [10]. The lesions in the Central Nervous System (CNS) are neuronal and/or neuropil vacuolation of the brainstem, mainly at the obex region, involving the dorsal motor nucleus of the vagus (DMNV) and also the spinal tract of the trigeminal nerve. Accumulation of the disease-associated abnormal prion protein (PrP^Sc^) is observed primarily in glial cells (astrocytes) and neurons [11,12]. The most susceptible alleles to CS are VRQ and ARQ and the most resistant ARR [13-16]. The biochemical signature of PrP^Sc ^is characterized by a three band pattern between 18 and 30 kDa [17].

Atypical/Nor98 scrapie (AS/Nor98), by contrast, seems to occur sporadically or to be minimally contagious [18]. In the majority of the cases, AS/Nor98 is detected only in a single sheep per flock [1,19,20]. It is distributed throughout a whole country [10,21] and its prevalence does not seem to vary through time [22]. AS/Nor98 agent has been shown to be experimentally transmissible, though at low efficiency, to sheep [23] and to transgenic mice expressing ovine [24] and porcine PrP [25]. However, its spread under natural conditions seems not to follow the same pattern as CS. Examination of British demographic factors and trading patterns has suggested that transmission of AS/Nor98 could occur, albeit at very low rate [26]. Two case-control studies of Nor98 in Norway and France found no risk factors to indicate transmission between flocks [21,27]. Neuropathologically, the PrP^Sc ^distribution pattern in the brain is characterised, in the majority of cases, by massive accumulations of PrP^Sc ^in cerebellum and cerebrum [1,28-32] and by the absence of deposition of PrP^Sc ^in the DMNV at the level of the obex [1,28,29,31-33]. Additionally, in some of the cases, PrP^Sc ^has been detected in the nucleus of the spinal tract of the trigeminal nerve [30,34,35]. Occasionally, the only presence of PrP^Sc ^immunostaining at the level of the obex appears to be a globular staining in the white matter tracts [36] while no intracellular immunolabelling in the central or peripheral nervous tissue has been observed in natural AS/Nor98 [31,37,38]. Genotypic features show that the PrP genotypes affected by AS/Nor98 are different from classical scrapie [10,20,33,35,39-43], the PrP alleles (136/141/154//171) ALHQ, AFRQ and ARR being the most frequently affected [36]. Biochemically, PrP^Sc ^of AS/Nor98 shows two characteristic features: one, the electrophoretic profile with a multiple band pattern and a characteristic band of low molecular weight (lower that 14 kDa), and the other one, a lower resistance to PK than PrP^Sc ^from CS [1,33,36,44].

Detection and identification of AS has been increasing since 1998, when Nor98 was first detected [1]. Various studies report AS/Nor98 cases in Belgium, The Falkland Islands, France, Germany, Ireland, Norway, Portugal, Sweden, UK, North America and Poland [1,19,29,32-35,39,45-50]. Besides, two recent studies based on active surveillance data from European countries report cases of AS in other countries such as Spain, Italy, Netherlands, Finland, Denmark and Switzerland [51,52]. In some of the countries where AS/Nor98 is reported, this form appears to be the most frequent if not the only one, as newly announced in New Zealand (media release appeared in http://www.biosecurity.govt.nz/media/28-10-09/atypical-scrapie-detection), and as observed in Poland where it constitutes the only small ruminant TSE detected [50].

In Spain, the proportion of AS/Nor98 cases increased considerably from 2% to 14% between 2004 and 2007 (*Report on the monitoring and testing of ruminants for the presence of transmissible spongiform encephalopathy (TSE) in the EU in 2007*) http://ec.europa.eu/food/food/biosafety/bse/annual_reps_en.htm. However, the monitoring requirements have changed during this period and it is highly probable that the testing and sampling methods have influenced the detection of these cases. Nevertheless, attention must be paid from now on to confirm whether AS/Nor98 tends to increase or remains constant along time.

The Latxa breed (called Manech in France) is the native breed of sheep in the Basque Country and it is bred for Idiazabal-type cheese production [53]. There are two varieties, black-faced Latxa and fair-faced Latxa that together constitute approximately 85% of the sheep population in the Spanish Basque Country [54,55].

Here we report molecular and neuropathological features of eight cases of AS/Nor98 detected between 2002 and 2009, and showing that AS/Nor98 is the most common small ruminant TSE form in Latxa breed sheep in the Spanish Basque Country.

## Case presentation

Between 2002 and 2008 the mean number of sheep analysed per year in the Basque Country was 764 and until September 2009, the total number of animals screened added up to 5620. Of these, eight Latxa ewes with molecular and pathological features of AS/Nor98 were found. The cases were detected widely distributed within this region and amounted to a significantly (p = 0.0196) higher prevalence (0.15%) than that of classical scrapie (0.02%) (Table 1). The first two cases were fallen stock and appeared in 2004, the third one was a slaughtered ewe tested in 2005 that was confirmed by Western blotting as Nor98 at the Norwegian National Veterinary Institute. The three following cases were detected in 2008, the first two cases at the beginning of the year and the third at the end of the year. The two last cases were detected at the beginning of 2009 and had been slaughtered for human consumption. Clinical symptoms were reported in only one of the cases (M31 (2008)). It drew the attention of the veterinary inspector at the slaughterhouse because it showed slight neurological signs such as ataxia, and poor condition. There were no further veterinary inspection reports of clinical signs for the remaining slaughtered animals or any of the fallen stock. In this context, it needs to be emphasized that due to frequent lack of clinical records as a consequence of the inefficiency of passive surveillance, there is no adequate clinical information on these scrapie cases in general. The mean age of all the eight cases was 7.9 years (range between 4.3 and 12.8).

**Table 1:** Distribution of small ruminants analysed and scrapie cases detected per year.

		Origin (%)				
				
Year	N	Fallen stock	Slaughtered	Others#	Atypical scrapie % (n)	Classical scrapie % (n)
2002	760	12.6	87.1	0.3	0	0
2003	1296	17.7	65.2	17.1	0	2.42 (26)^c^
2004	761	32.5	61.9	5.6	0.28 (2)^a^	0
2005	503	40.9	59.1	0.0	0.19 (1)^b^	0
2006	710	27.0	73.0	0.0	0	0
2007	710	18.3	81.7	0.0	0	0
2008	610	15.1	84.9	0.0	0.49 (3)^a, b^	0
2009*	270	23.3	76.7	0.0	0.74 (2)^b^	0
Total	5620				0.14 (8)	0.02 (1)

N: total number of small ruminants analysed. (#) Others include clinical suspects and animals killed for eradication purposes. Atypical and classical scrapie annual rates were calculated over the sum of fallen stock and slaughtered animals.

^a ^Fallen stock. ^b^Slaughtered. ^c ^Outbreak in one flock.

*January to September.

**Figure 1:**
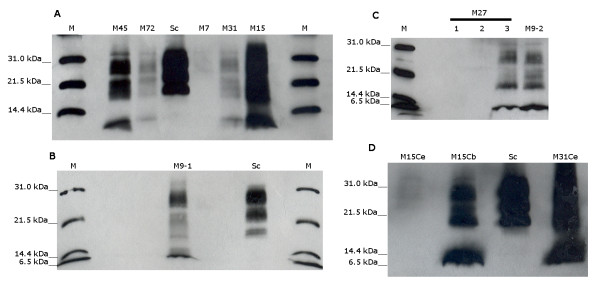
**Immunoblot with TeSeE Western Blot**. A) Obex region of cases M45, M72, M7, M31 and M15. B) Cerebellum of case M9-1. C) Ten-fold concentrated samples from obex (1), cerebellum (2) and cortex (3) of case M27, and to obex of case M9-2. D) Cerebellum and cerebrum (cortex) of case M15 and cerebellum of case M31 (M31Ce). *Sc*: Classical scrapie control. *M*: Molecular weight marker.

Results of rapid test, immunoblot analyses and genotyping are shown in detail in Table 2. The homogenates originally tested by rapid tests were made from a pool of obex and cerebellum tissues and the optical densities varied from 0.363 to 2.859. Immunoblot analyses with Prionics Check Western revealed that 6H4 monoclonal antibody failed to detect abnormal prion protein in all cases. We replaced this antibody by P4 mAb and obtained then a very weak signal (data not shown). The best signal in western blot was achieved with TeSeE Western blot (Bio-Rad), where the PrP^Sc ^profile showed the characteristic multiband pattern clearly different from those of the classical scrapie cases (Figure 1). The analysis of different brain regions (when possible) by TeSeE Western blot revealed the strongest signal from the cerebellar tissues for all the cases except for M27 (Figure 1C) and M15 (Figure 1D), where the cerebrum presented a more intense PrP^Sc ^signal than the cerebellum. Although case M7 showed an extremely faint PrP^Sc ^signal in the obex region and no staining in the cerebellum, the case was positive in the rapid test, and then it was confirmed positive by immunohistochemistry at the National Reference Centre for TSE in Zaragoza.

**Table 2:** Results of rapid test (ELISA TeSeE), immunoblot (TeSeE Western Blot), IHC (cocktail of 2G11 and F89/160.1.5) and PrP genotypes of AS cases.

	2004		2005	2008			2009	
	M7	M72	M45	M31	M15	M19-1	M27	M9-2
**Origin**	Fallen stock	Fallen stock	Slaughtered	Slaughtered	Fallen stock (Advanced autolysis)	Fallen stock	Slaughtered	Slaughtered
**Month of death**	June	November	April	January	February	December	February	April
**Province**	Vizcaya	Alava	Guipuzcoa	Guipuzcoa	Alava	Guipuzcoa	Guipuzcoa	Guipuzcoa
**Age (years)**	6.3	12.8	4.3	7.0	9.1	5.6	8.1	10.3
**Genotype**	AFRQ ALRQ	ALRR ALRQ	AFRQ ALRQ	AFRQ AFRQ	AFRQ AFRQ	AFRQ ALHQ	ALRQ ALRH	ALRQ ALRQ
**Clinical symptoms**	Not registered	Not registered	Not registered	Yes	Not registered	Not registered	Not registered	Not registered
**Rapid test result** **(cut off)**	0.377 (0.218)	0.387 (0.219)	1.112 (0.219)	2.788 (0.230)	2.859 (0.230)	2.710 (0.219)	0.498 (0.102)	1.289 (0.103)
**WB TeSeE**								
**Obex-Cerebellum**	1	2	3	2	3	1	1	2
**Cerebellum**	0	0	3	3	1	3	1	3
**Cerebral Cortex**	0	0	1	0	2	1	1(3)*	2
**IHC**								
**MO**	PC	NA	NA	2	1	1	1	1
**Cerebellum**	1	3	3	3	1	3	1	3
**Frontal Lobe**	PC	NA	2	NA	3	1	2	1

Numbers on WB column indicates the signal intensity according to Figure 1. 0: negative, 1: faint, 2: moderate and 3: intense. NA: Not available. PC: Poor condition. MO: Medulla oblongata. Immunohistochemical staining was graded subjectively for intensity and spread from 0 to 3 as in WB. * Refers to PrP^Sc ^signal after concentrating the sample 10 folds.

PrP genotyping showed variability in genotypes. All animals were AA_136 _and no VV_136 _alleles were found. AF_141_RQ was the most frequent (7/16) allele followed by AL_141_RQ (5/16). Moreover, in combination with wild type (ALRQ), a single allele of AL_141_RR and AL_141_RH was also found. A novel AS allele involvement was found for case M9-1, which had allele AL_141_HQ; however this was combined with F141 in the other allele which is a susceptibility codon for AS.

**Figure 2:**
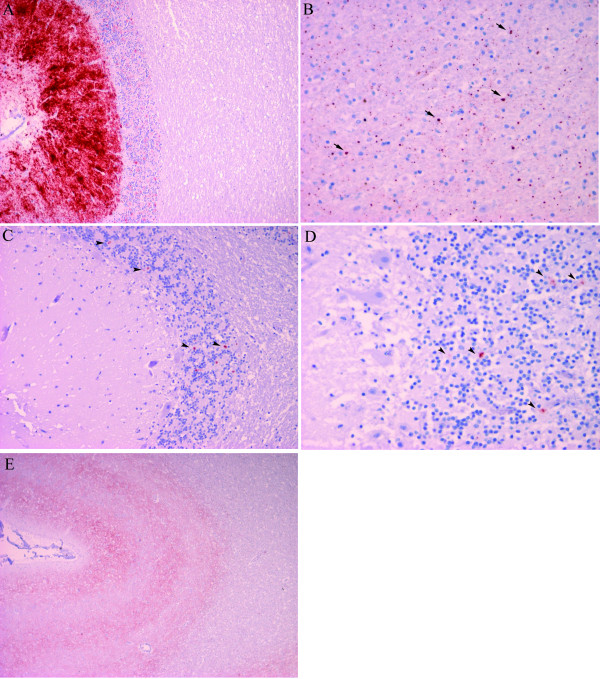
**Immunohistochemistry for PrP^Sc ^using a cocktail of mAb F89/160.1.5 and 2G11**. A) Cerebellum of case M72 (100×) showing intense immunostaining in the molecular layer of cerebellar cortex. B) Frontal lobe of case M27 with F89/160.1.5/2G11, (400×) showing punctate globular immunostaining (arrow) in white matter. C) PrP^Sc ^deposits (arrowheads) in the granular layer of the cerebellum of case M27. Scant fine granular deposits of PrP^Sc ^visible in the granular layer (200×). D) Cerebellar cortex of case M15 with scant fine granular (arrowheads) deposits of PrP^Sc ^visible in the granular layer (400×). E) Cerebral cortex of case M15 (40×) showing fine granular immunolabelling following a laminar pattern.

Immunohistochemical analyses were performed in the cerebellum, frontal lobe and medulla oblongata with a mix of mAb F89/160.1.5 and 2G11. In all five cases where the cerebellum was available (M72, M45, M31, M9-1 and M9-2), this was the brain region where PrP^Sc ^deposits were more abundant. The type of deposits were rather homogeneous and diffuse, predominating the fine granular staining in the cortical layers and with the molecular layer showing a more prominent signal than the granular layers (Figure 2A). Punctate aggregates, plaque-like and linear deposits were also present in the molecular layer. The cerebrum of three available cases showed punctate staining in the deeper cortex (M45 and M9-1) and a fine granular laminar pattern (M9-2). Basal nuclei, when present (M45) showed intense punctate and fine granular signal. In the medulla oblongata the deposits were punctate and fine granular in the neuropil of nuclei, especially in the spinal tract of trigeminal nerve. In the three different areas, the white matter showed variable degrees of punctate, globular aggregates, or plaque like PrP^Sc ^deposits. In the cerebrum, this signal in the white matter was in some cases (M45 and M9-1) more prominent than in the grey matter.

Remarkable differences were found in M15 and M27 cases in which the sample from frontal cerebrum showed the strongest signal (Figure 2B) and the cerebellar immunolabelling pattern was dissimilar comparing to other AS cases. The immunostaining of cerebellum of both cases showed scant deposits in the granular layer but more prominent than in the molecular (Figure 2C and 2D). In the frontal lobe, M15 showed fine granular staining in a three band laminar pattern in cortex (Figure 2E), whereas in M27 the deeper layers of the cortex showed punctate immunostaining. The basal nuclei showed punctate pattern when present (M15). No differences were found in the medulla oblongata in relation with the rest of the cases. Apart from these two cases, M7 sample was in poor condition and showed scant immunostaining in the granular layer of the cerebellum. This result could be related with an excessive tissue fixation (during 5 years in this case) of the sections as recently observed when stained with different antibodies like mAb 2G11 and F89/160.1.5 [56].

## Discussion

We described eight atypical scrapie cases detected between 2002 and 2009 from the Basque Country. All AS/Nor98 cases were found in Latxa sheep which breed represents 85% of the Basque Country sheep population [54,55,57]. The occurrence of these cases seemed to be random and, in agreement with other AS surveillance studies [22], there was no apparent temporal trend. Geographically, the distribution of atypical scrapie cases was in accordance with that described in other regions of the world. First, a single positive sheep per affected flock was detected, as observed in the majority of other AS cases [1,19,20]. Second, they presented a wide distribution in the Basque Country, with reports of cases in all three provinces. At this point it should be mentioned that the highest proportion was observed in Guipuzcoa (62.5%) but it may be due to the fact that it constituted the province with the highest rate of sheep slaughter (over 90%) analysed in the Basque Country. Nevertheless, the sample size was still too small to draw any definite conclusion. Moreover, the occurrence of atypical scrapie cases pointed to an absence of time clustering, since there were long periods with no detection of cases and then, within a few months 2 or 3 affected animals were detected. However, it must be taken into consideration that, (i) not all sheep older than 18 months of age were analysed as a consequence of the random sampling procedure contrary to the exhaustive one legislated for cattle, (ii) the brain sampling may not have been optimal, e.g. only the medulla oblongata was collected or, particularly in the case of fallen stock, the brain samples were sometimes severely autolytic and liquefied, thus increasing the chances of sampling an area where PrP^Sc ^was absent, (iii) due to the young age of some animals, the stage of the disease, the small sampling site and the relatively low number of animals and short period of time involved, the possibility of longer and more tenuous temporal and spatial trends in PrP^Sc ^distribution could not be excluded. For these reasons, the number of AS and CS cases may be underrepresented and could suffer from a certain bias.

One of the cases (M45) described here was confirmed to be Nor98. PrP^Sc ^deposition, distribution and molecular profile of the cases M72, M31, M15, M9-1 and M9-2 were identical to the features of this Nor98-confirmed case and to previous descriptions [1,38]. Moreover, the mean age observed was in accordance to other observations for atypical scrapie [36]. Among some of the features our cases had in common with M45, the following should be emphasised: i) the molecular protein profile showed a characteristic low molecular weight band under 14 kDa, ii) the cerebellum was the most affected region, iii) PrP^Sc ^was mainly detected in the neuropil predominantly as fine granular deposits, and iv) a faint to moderate PrP^Sc ^signal intensity was seen. The detection of more intense PrP^Sc ^deposits in cerebellum or cerebral cortex rather than in the medulla oblongata may indicate that the prion is likely not to enter the brain through the medulla (DMNV) as described for classical scrapie [58], thus suggesting a rather sporadic aetiology, as observed in human sporadic TSE cases. Cases M15 and M27 however, presented some differences. Albeit PrP^Sc ^molecular pattern was similar to the Nor98 confirmed case, both animals showed more PrP^Sc ^deposits in the frontal lobe of cerebral cortex than in the cerebellum by immunohistochemistry (IHC) and also by immunoblot (WB) for case M27. By contrast, case M15 showed small differences between IHC and WB results since the signal in the pooled obex and cerebellum in WB was more intense than in the cerebellum and medulla oblongata in IHC. This could have been biased by the sampling for frozen tissues and by severe tissue autolysis and could be the explanation for a negative and extremely faint PrP^Sc ^signal in WB of case M7 in the cerebellum and obex, respectively. The fact that these cases showed more PrP^Sc ^accumulation in cerebral cortex than in cerebellum might be influenced by other still unknown environmental or genetics factors. Alternatively, this might happen more commonly than observed because of the limited number of AS/Nor98 cases where both the cerebellar and the cerebral cortices are available for analysis. When the sampling of brain is carried out with a spoon through the foramen magnum some cerebellum can also be collected along with the medulla oblongata and this can be targeted as the optimum sample for WB testing, particularly if the IHC results indicate a possible atypical scrapie case. Unfortunately, cerebrum is not routinely collected by this method so little is known about its PrP^Sc ^status. The availability of this brain region for M7 would have been useful to clear up any doubt on its diagnosis and classification. This case was questionable because we did not obtain a clear pattern in the WB with the band lower than 14 kDa size and because the detected signal in the cerebellum by means of IHC was extremely faint. The poor condition of the sample and the scant material available did not allow us to obtain, after repetitions of the analyses, a clear evidence of being an AS/Nor98 case. The fact that it was confirmed for the National Reference Laboratory allowed arguing that it was a scrapie case. Besides, there were several features supporting it as an atypical scrapie case. First, for CS, we would have expected to obtain a more intense signal in the WB and a clear three bands pattern of PrP^Sc ^in the region of the obex [17]. Second, even not having an optimal signal in the WB that showed the lower band characteristic of atypical cases and having evidences according to which the case was positive by means of IHC and rapid test, the possibility of an atypical scrapie case should not be excluded. In this case, it could have happened that there was little amount of abnormal PrP^Sc ^so that after PK digestion the amount of resistant PrP^Sc ^was reduced considerably below the detection threshold of WB, as observed in the cerebellum of case M15. Third, the age of this sheep was higher than the mean age of CS cases described in Latxa breed [59] and in other breeds [60,61]. Finally, this was the only detected case in its herd, which was in agreement with the epidemiology of the AS/Nor98 [1,19,20].

The majority of PrP genotypes described herein were observed in other AS/Nor98 cases [36]. We found an over-representation of animals carrying AF_141_RQ and AL_141_RQ alleles, suggesting that these alleles may confer more susceptibility to atypical scrapie in Latxa breed sheep. We also described a novel genotype associated to AS that has not been previously described (AL_141_RQ/AL_141_RH).

Case reports from this study and other case reports from Spain http://www.eeb.es/pags/espana.htm and Portugal [35], indicate a high frequency of atypical cases compared to CS outbreaks in the Iberian Peninsula. It could be speculated that AS/Nor98 is the traditional form of scrapie in the Iberian part of the Basque country, whilst in the French part, the classical form has been predominant [59]. This would point to some unidentified epidemiological features limiting the spread of classical scrapie in the Iberian Peninsula. However, since the analysis of all sheep can not be guaranteed, it is difficult to test this hypothesis. The analysis of all sheep would provide more information about the epidemiology and pathology of this disease. Moreover, it could contribute in assessing whether AS is present in the Basque Country with the same high frequencies as others human TSEs, such as sporadic Creutzfeldt-Jakob disease [60] and Fatal Familiar Insomnia compared to other Spanish autonomous communities (National Epidemiology Centre: http://www.isciii.es/htdocs/centros/epidemiologia/epidemiologia_listado_ecj.jsp).

## Conclusions

This work indicates that AS/Nor98 constitutes the most common small ruminant Transmissible Spongiform Encephalopathy form in the Latxa breed in the Spanish Basque Country, where it also affects a genotype (ALRQ/ALRH) not previously associated to this form of TSE.

## Methods

### Animals and Tissues

Heads from slaughtered and fallen stock small ruminants were received at Neiker-Tecnalia. During 2002 to 2004 obex and thereafter both obex and cerebellum tissues were collected with a specifically designed sampling spoon. Sampled heads were conserved refrigerated until the results of rapid test were obtained. Portions of cerebral frontal lobe, cerebellum and medulla oblongata of positive samples were frozen at -20°C for immunoblotting analysis and other portions fixed in 10% buffered formalin prior to be embedded in paraffin according to standard procedures for immunohistochemical analysis. Four μm sections of paraffin blocks and frozen tissue were sent to the National Reference Laboratory (from 2002 to 2005 to Zaragoza, and since 2006 to Algete). In some cases, not all three brain levels were available due to autolysis or sampling problems. This protocol is part of the TSE prevention and control programme of the Basque Country.

### Rapid test

In order to detect PrP^Sc ^in small ruminants, Bio-Rad TeSeE™ ELISA rapid test was used following manufacturer's recommendations.

### Immunohistochemistry

The sections were treated with formic acid for 30 min and autoclaved at 121°C in 0.01 M citric acid, pH 6.1 for 30 min. Then they were submitted to a light PK digestion (4 μg/ml) at 37°C for 5 minutes prior to immunostaining using a cocktail of mAb F89/160.1.5 (VMRD, Washington, USA) and 2G11 (Institute Pourquier, France) and EnVision+ System, HRP Peroxidase-AEC (3-amino-9-ethylcarbazole) kit (DakoCytomation, California, USA). Immunostained sections were briefly counterstained with a haematoxylin solution and mounted with aquamount gel. Definition of PrP^Sc ^deposits were performed according to a previous publication [38].

### PrP Biochemical analysis: immunoblot

Molecular characterization was performed using Prionics Check Western SR (Prionics) modified [61] and TeSeE Western blot (Bio-Rad). The modification of Prionics WB protocol involved the replacement of mAb 6H4 (aa 147-155, [62]) by P4, directed against residues 93 to 99 [63,64] of the ovine PrP. TeSeE Western blot protocol was performed following manufacturer's recommendations as described previously [39].

### PRNP Genotyping

DNA from brain samples was obtained using QIAamp DNA Mini Kit (QIAGEN). Genotyping of PrP polymorphisms at codons 136, 154 and 171 was initially carried out by real time PCR on an ABI PRISM 7000 (Applied Biosystems) as previously described [65] at NEIKER-Tecnalia. In addition, analysis of these codons, as well as codon 141, was carried out at the Norwegian School of Veterinary Science Oslo by DNA sequencing. The samples were amplified with the forward primer 5' AGGCTGGGGTCAAGGTGGTAGC and reverse primer 5' TGGTACTGGGTGATGCACATTTGC modified by 5' attachment of M13-21 and M13 rev tails allowing the use of commercially available fluorescence labelled primers and then sequenced using Big Dye Primer chemistry (Applied Biosystems). The polymorphisms were identified by manual inspection of the sequence electropherograms.

### Statistical analysis

Comparison of the incidence of classical and atypical scrapie during the period of study was performed with the Fisher exact test of the FREQ procedure in the SAS statistical package (SAS Insitute, Inc., Cary, NC, USA).

## Authors' contributions

ABRM carried out the molecular analyses, participated in the genotyping and data collection and drafted the manuscript. JMG participated in the sampling and data collection and participated in the design of the study and coordination and helped to draft the manuscript. SM, LB and MG contributed to sampling, performing of rapid test and genotyping. NG and EM carried out initial histopathological and immunohistochemical processing and interpreted the results. SLB carried out the immunohistochemical analysis of the sections with F89/160.1.5 and 2G11 and supervised the genotyping of codon 141. RJU is the head of the project and had primary responsibility in the design and supervision of the investigations reported here as well as of writing of the manuscript. All authors read and approved the final manuscript.
